# Starvation reduces thermal limits of the widespread copepod *Acartia tonsa*

**DOI:** 10.1002/ece3.10586

**Published:** 2023-10-03

**Authors:** Gaia Rueda Moreno, Matthew C. Sasaki

**Affiliations:** ^1^ Deparment of Biology New York University New York New York USA; ^2^ Department of Marine Sciences University of Connecticut Groton Connecticut USA; ^3^ Department of Biology University of Vermont Burlington Vermont USA

**Keywords:** climate change, copepod, starvation, temperature, thermal limit

## Abstract

Organismal thermal limits affect a wide range of biogeographical and ecological processes. Copepods are some of the most abundant animals on the planet and play key roles in aquatic habitats. Despite their abundance and ecological importance, there is limited data on the factors that affect copepod thermal limits, impeding our ability to predict how aquatic ecosystems will be affected by anthropogenic climate change. In a warming ocean, one factor that may have particularly important effects on thermal limits is the availability of food. A recently proposed feedback loop known as “metabolic meltdown” suggests that starvation and exposure to high temperatures interact to drastically reduce organismal thermal limits, increasing vulnerability to warming. To investigate one component of this feedback loop, we examined how starvation affects thermal limits (critical thermal maxima: CT_max_) of *Acartia tonsa*, a widespread estuarine copepod. We found that there was no effect of short‐duration exposure to starvation (up to 2 days). However, after 3 days, there was a significant decrease in the CT_max_ of starved copepods relative to the fed controls. Our results provide empirical evidence that extended periods of starvation reduce thermal limits, potentially initiating “metabolic meltdown” in this key species of coastal copepod. This suggests that changes in food availability may increase the vulnerability of copepods to increasing temperatures, amplifying the effects of climate change on coastal systems.

## Graphical abstract

Environmental conditions and the availability of food can interact to affect organismal performance. This study examines the effect food limitation has on upper thermal limits of a widespread copepod, *Acartia tonsa*. We find that copepods are resistant to short periods of starvation, but thermal limits are severely reduced after several days.

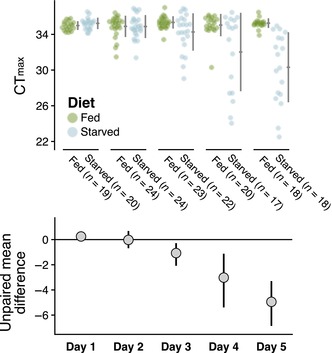

## 1 INTRODUCTION

The acquisition of nutrition is a fundamental challenge for organisms. Environmental conditions have well‐known effects on the ability to find, capture, and ingest food via a direct influence on organismal performance. Temperature, for example, has strong effects on metabolic rates in ectothermic organisms, linking both performance and energetic requirements to the thermal environment (Brown et al., 2004; Johnston et al., 2022). As energetic requirements tend to increase with warming, we might expect to see increasing consumption pressure on autotrophs and lower trophic levels as ocean warming proceeds, with consequences for community structure and the distribution of biomass (Archibald et al., 2022). Nutrition may also affect how environmental conditions impact organisms, however, by modifying the underlying sensitivity to changes in the experienced environment (Huey & Kingsolver, 2019; Litchman & Thomas, 2023). As anthropogenic climate change is driving long‐term ocean warming and increased frequency of disturbances like marine heat waves (Harvey et al., 2022; Hobday et al., 2016; Johnston et al., 2022; Li & Donner, 2022; Oliver et al., 2018; Smale et al., 2019), these feedbacks between feeding and sensitivity to environmental conditions are crucial to consider.

The interactions between starvation and upper thermal limits are still unknown for many taxa. Most studies have focused on terrestrial arthropods, examining species in Diptera (Gotcha et al., 2018; Kalra et al., 2017; Manenti et al., 2018; Mitchell et al., 2017), Coleoptera (Chidawanyika et al., 2017; Scharf et al., 2016), Hymenoptera (Gonzalez et al., 2022; Nguyen et al., 2017), Hempitera (DeVries et al., 2016), and Lepidotera (Mir & Qamar, 2018; Mutamiswa et al., 2018). There is strong variation in the effects of starvation on thermal limits across this body of work. The duration of starvation is likely important to consider, with little to no effect of shorter periods of starvation (DeVries et al., 2016; Gonzalez et al., 2022). However, even relatively short exposures have been observed to reduce thermal limits in some insect taxa (Manenti et al., 2018; Mir & Qamar, 2018). Further, a number of studies have reported that starvation actually increases thermal limits (Bubliy et al., 2011; Gotcha et al., 2018; Kalra et al., 2017), possibly due to starvation‐induced changes in energy allocation. Within aquatic ectotherms, experiments on the freshwater amphipod *Gammarus fossarum* revealed that starvation improved survival during acute heat stress (Semsar‐kazerouni et al., 2020). This improvement in thermal limits is not observed in fish, octopus, or damselfly larvae (Dinh et al., 2016; Lee et al., 2016; Uriarte et al., 2018).

The species‐specific nature of starvation effects on thermal limits is an important observation for predictions about the response of communities to climate change. Amplifying the direct effects of starvation on thermal limits, Huey and Kingsolver (2019) describe a process termed ‘metabolic meltdown’, in which exposure to high temperatures, reduced food intake, and decreasing thermal limits act synergistically to drastically decrease tolerance to a warmer environment. Since a key component of metabolic meltdown is the reduction of thermal limits under reduced food intake, variation in starvation effects across taxa may determine the relative vulnerability of community members to events like heatwaves, thus shaping community dynamics in a changing climate. These dynamics are important to consider, as compound events (anomalously high temperature co‐occurring with low productivity) are fairly common in marine systems, especially in low latitude waters and in the Southern Ocean (Le Grix et al., 2022).

Copepods are the some of the most abundant animals on the planet, and dominate planktonic communities in the coastal ocean (Turner, 2004). By nature of their abundance, this group plays key ecological and biogeochemical roles in aquatic systems (Brun et al., 2019; Pinti et al., 2023; Steinberg & Landry, 2017). In particular, copepods are important consumers of primary productivity, and act as a crucial linkage between phytoplankton and higher trophic levels (Castonguay et al., 2008). Climate change, therefore, has the potential to impact aquatic ecosystem functions as well as human fishery systems directly through effects on copepod populations. However, despite their abundance and ecological importance, there is limited data on environmental control of copepod thermal limits, including how their thermal limits are affected by starvation. This impedes our ability to predict how copepod populations may be affected by co‐occurring changes in temperature and food availability over both short (e.g., seasonal changes) and long timescales (e.g., anthropogenic climate change). *Acartia tonsa* is an abundant and widespread calanoid copepod and plays an important role in coastal food webs (Turner, 1981, 2004). In this study, we tracked changes in critical thermal maxima (CT_max_) in the widespread copepod *A. tonsa* during extended starvation to test the hypothesis that food deprivation reduces thermal limits.

## 2 METHODS

### 2.1 Copepod cultures

The copepods used in this study were collected in July 2020 from Esker Point, Connecticut (41.3206 N, −72.002 W) by surface tow with a 63‐μm mesh net and solid cod end. Mature *A. tonsa* females and males were isolated from the two contents and used to initiate a laboratory culture, which was maintained in 12 qt buckets. Cultures were kept in an environmental chamber at 18°C with a 12:12 light:dark cycle. Once established, these cultures typically contained around a thousand individuals. Large cultures such as these appear to maintain high levels of genetic diversity even after long periods maintained under laboratory conditions (Dam et al., 2021). A small aquarium pump ensured constant aeration. Copepods were transferred to 0.2 μm filtered seawater (salinity ~30 practical salinity units) on a weekly basis. Copepods were fed ad libitum a mixture of three phytoplankton cultured in F/2 media under the same environmental conditions: a green flagellate, *Tetraselmis* sp.; a cryptomonad *Rhodomonas* sp.; and a small diatom, *Thalassiosira weissflogii*. This diet is regularly used to maintain large, active laboratory cultures of *A. tonsa* (Sasaki & Dam, 2021a).

### 2.2 Measuring thermal limits

The experiments described here were performed during the summer of 2022. Information about the specific experimental design (sample sizes, diet treatments, individual sexes, etc.) is provided in Section 2.3. We used a custom setup to measure critical thermal maxima (CT_max_) of individual copepods. Details of this method are provided in Sasaki et al. (2023). Briefly, this set up includes a reservoir, water bath, and temperature logger. The reservoir (a five‐gallon bucket) holds ~15 L of water, along with a 300‐watt fixed output titanium water heater and two aquarium pumps. One pump vigorously circulates water within the reservoir while the other pumps water up into the water bath, a plexiglass tank that sits atop the reservoir. The water bath contains a series of test tube holders, used to position the experimental vessels (50 mL flat‐bottom glass tubes) during the assay. When the pump is turned on, water floods the bath and then spills over back into the reservoir. In this arrangement, temperatures in the experimental vessels are slowly increased at a rate of between 0.1 and 0.3°C/min, following temperatures in the reservoir. The final component is a small Arduino logger, which records the temperature from three sensors placed inside tubes distributed throughout the water bath.

At the beginning of each CT_max_ trial, the water in the reservoir was adjusted to 18°C. The experimental vessels were filled with 10 mL of 0.2 μm filtered seawater before being placed in the water bath, which when flooded brought the experimental vessels to the correct temperature as well. Individual copepods were placed into the vials (*n* = 10 per assay), and let rest for 10 min at constant temperature. Only mature females were used in these experiments. All copepods were checked during this time period for normal behavior. Individuals exhibiting abnormal behaviors were excluded from further analysis. In this case, abnormal behavior was defined as an individual laying on the bottom of the vial and not responding to gentle stimuli. After this resting phase, the water heater was turned on, initiating the temperature ramp. Simultaneously, the temperature logger began to record temperature and a stopwatch began recording the time passed. Individuals were continuously monitored as water temperature increased. CT_max_ is generally defined as the temperature at which an individual ceases to respond to physical stimuli (Cowles & Bogert, 1944), indicating the onset of “ecological death” (the inability to escape lethal temperatures, predators, etc.). In *A. tonsa* this is indicated by cessation of movement, a lack of response to gentle physical stimuli (e.g., slow flushing of the water in the tube with a transfer pipette), and abnormal body configuration (specifically—antennules pressed against the sides of prosome and a distinct dorsal tilt of the urosome). The time at which an individual began to exhibit these characteristics was recorded and then that individual's experimental vessel was removed from the water bath. After all individuals reached their CT_max_, copepods were photographed using a camera attached to an inverted microscope, and body size was estimated using a scale micrometer and the software ImageJ (Schneider et al., 2012).

The times recorded during the trials were converted to CT_max_ values in degrees C using the temperatures recorded on the Arduino logging system. As there are between 1 and 10 vials being monitored at any point in the trial, the time at which an individual was recorded as having stopped responding to stimulus corresponds with the latest time (and, therefore, highest temperature) it could have reached its CT_max_. The period of time during which an individual could have reached its CT_max_ extends from this definite end point to the last time the individual was checked. As it generally takes around 5 s to check whether an individual has stopped responding, the duration of this period of time was estimated for each individual as the number of vials remaining in the water bath multiplied by 5 s. As a result, this uncertainty window decreased in length as the trial went on, until, for the final individual, the window includes just the amount of time it took to check whether the individual had stopped responding. For each individual, CT_max_ is estimated as the average temperature recorded by all three temperature sensors throughout the uncertainty window. We used this time‐based method instead of directly monitoring the temperatures because (i) it was more efficient to record the time than the temperature readings from three separate sensors, and (ii) to reduce any sub‐conscious bias stemming from past knowledge or expectations about the copepod thermal limits.

### 2.3 Experimental design

**FIGURE 1:**
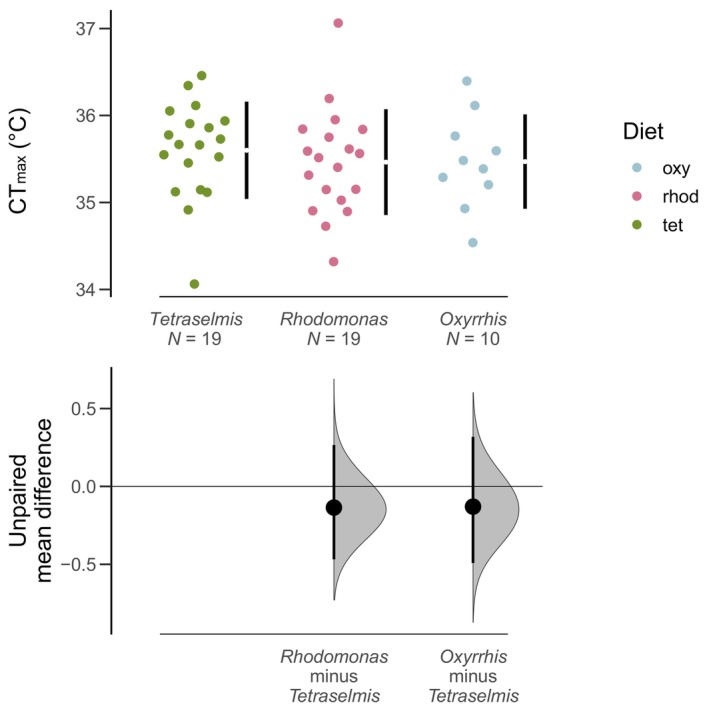
Estimation plots showing the effect of different diet treatments on thermal limits. Measurements were taken after 24 h, and show that thermal limits were not altered by short exposure to the different diets.

We used five replicate experiments to test our hypothesis that starvation reduces copepod thermal limits over time. Each experiment involved measuring a baseline CT_max_ for the culture, and then making daily CT_max_ measurements for two groups of copepods, a fed control group and a starved treatment group. To initiate each experiment, 10 mature females were isolated from the laboratory culture and maintained for 24 h in 200 mL of an ad libitum food solution (800 μg C/L of *Tetraselmis*). Preliminary work showed that short exposure to three different prey options (*Tetraselmis* sp., *Rhodomonas* sp., and *Oxyrrhis marina*, a heterotrophic dinoflagellate) did not affect copepod thermal limits (Figure 1). After 24 h, CT_max_ was measured as described above. This provided an initial baseline value for each experiment.

On the same day as the baseline CT_max_ measurements, ~90 mature females were isolated from the culture and divided into six groups. The six groups were randomly assigned to either the starvation treatment or the fed control treatment. The starvation groups were maintained in 0.2‐μm filtered seawater. The control group was provided with the food solution used prior to measuring baseline CT_max_ (800 μg C/L of *Tetraselmis*). Each group was kept in a 100 mL cup with a plastic cylinder nested within. The bottom end of the cylinder was covered by a 150‐μm mesh screen. Similar set‐ups are often used to prevent egg cannibalization during egg production assays, as eggs of *A. tonsa* sink through the mesh to the base of the cup (Plough et al., 2018). In our case, this prevented females in the starvation group from acquiring nutrition via egg cannibalism. All groups were transferred to fresh media (either filtered seawater or food solution) on a daily basis throughout the experiment by gently removing the meshed column and placing it into a new 100 mL cup.

Copepods from these six groups were then used to measure thermal limits each day for 5 days, starting 24 h after the females were first isolated. Each day, we measured CT_max_ values for 10 copepods, selected at random from the six groups (see the accompanying code for the randomization script used). By repeating these measurements over the 5‐day period, we were able to examine how the effects of starvation on CT_max_ changed as the duration of exposure increased. The second replicate experiment was ended after day three when all individuals from the starvation treatment died. Individuals from replicate experiment five were not photographed after the CT_max_ assays due to a malfunction with the imaging software.

### 2.4 Statistical analysis

All statistical analyses were performed with R (Version 4.1.3; R Core Team 2022). We examined the effects of starvation using mean difference as an effect size estimate. Confidence intervals for this effect size were estimated using non‐parametric bootstrapping (Ho et al., 2019). Two sets of effect sizes were estimated. First, we examined the difference between the initial baseline CT_max_ values and CT_max_ values for fed and starved individuals during the experiment (e.g., the difference between fed CT_max_ and the baseline CT_max_ on each of days 1 through 5). The second effect size was the difference between fed and starved individuals on each day within the experiment. If CT_max_ does not change over time in the fed control group, these two effect sizes will provide similar results.

## 3 RESULTS

**FIGURE 2:**
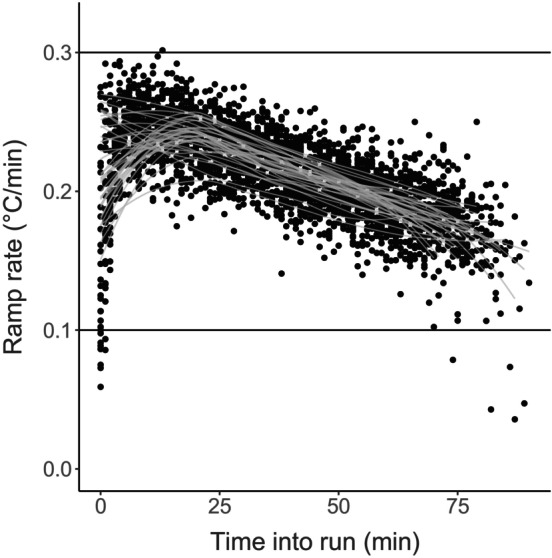
A scatterplot of the observed ramping rates (the increase in temperature per minute) during the CT_max_ trials. Each point represents the average ramping rate over a period of 1 min. A loess smoother is included for each individual experiment. Ramping rates were generally between the target values of 0.1 and 0.3°C/min, and consistent across experiments.

A total of 254 CT_max_ measurements were made across the five replicate experiments (153 from the fed controls and 101 from the starved treatment). Note that the difference between the number of fed and starved individuals reflects the five sets of initial baseline CT_max_ values measured (all experienced ‘fed’ conditions). A total of 16 individuals were excluded for abnormal behavior (~0.6% of individuals). These individuals came from both the fed and starved treatment, and we, therefore, do not believe this behavior to be related to diet treatment, but instead may have been due to damage during the transfer process. The custom setup produced consistent ramping rates across assays (Figure 2). Ramping rates did, however, decrease over time in a consistent way within each assay due to the imperfect insulation of the bucket reservoir. Nonetheless, ramping rates were always between the target ramping rates of 0.1–0.3°C/min, which have been used previously to measure CT_max_ for copepods (Harada et al., 2019; Jiang et al., 2009; Sasaki & Dam, 2021b).

**FIGURE 3:**
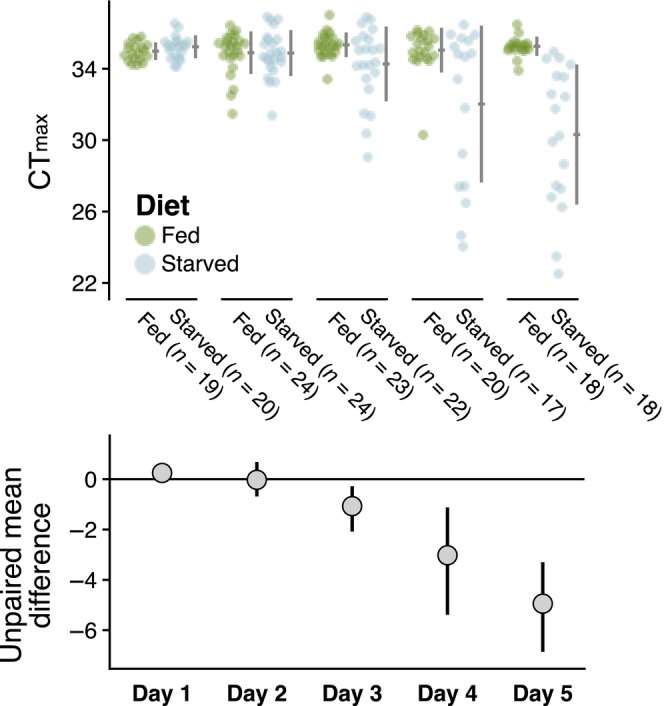
Estimation plots depicting the gradual reduction in thermal limits (measured as CT_max_) in the starvation group relative to the fed control group. The top panel shows the raw CT_max_ values, with fed and starved individuals in green and blue, respectively. The bottom panel shows the calculated effect sizes for each comparison, along with 95% confidence intervals.

Thermal limits of the fed control individuals were consistent with the baseline CT_max_ values across the entire experiment. We will, therefore, focus on the direct comparisons between fed controls and starved individuals. Mean CT_max_ in the starved group gradually decreased over the course of the 5‐day starvation period relative to the fed controls (Figure 3).

**FIGURE 4:**
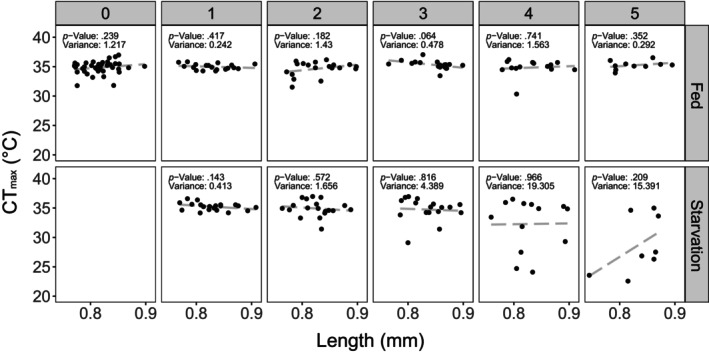
Thermal limits (CT_max_) plotted against individual body size for each day, separated by treatment. A linear regression is shown, along with the associated *p*‐value for each day. Thermal limits are not correlated with body size on any of the experimental days, indicated by the dashed regression. The variance in CT_max_ is also included for each day and treatment group.

There was no difference between thermal limits of fed and starved individuals on days 1 or 2. Thermal limits were approximately 1°C lower by day 3 and continued to decrease by approximately 2°C/day after that. By day 5, starved individuals had thermal limits that were approximately 5°C lower than those of control individuals. There was, however, also a large increase in the variance of individual thermal limits in the starved treatment; several individuals maintained thermal limits similar to those observed in the control individuals while others had thermal limits as low as 22°C. There was no significant effect of body size on CT_max_ during any of the experimental days, despite substantial size variation (Figure 4). We note, however, that a negative trend was observed on day 5 in the starvation treatment, which may be worth further investigation.

## 4 DISCUSSION

Marine biota are threatened by both ongoing warming and changes in food quality and quantity. Examining the interactions between starvation and environmental sensitivity may, therefore, provide useful insights into the responses of marine communities to climate change. We tested the hypothesis that food deprivation reduces thermal limits in the important coastal copepod species *A. tonsa*. Using a series of replicated laboratory experiments, we show that the upper thermal limits of *A. tonsa* are substantially reduced after just a few days without food, although this reduction was highly variable across individuals.

Previous studies have shown a wide range of starvation effects on upper thermal limits. The range of responses implicates several potential mechanistic bases starvation effects on thermal limits. In systems where thermal limits increase due to starvation, enhanced lipid accumulation at the initiation of starvation and changes in energetic allocation have been suggested as a mechanistic basis (Djawdan et al., 1998; Klepsatel et al., 2016; Sokolova, 2013). Here, however, we observed a decrease in thermal limits resulting from starvation. In other systems where this has been observed, the depletion of energetic reserves and the accompanying reduction in metabolic rates are often suggested as a potential physiological basis. Starvation tolerance in copepods may be mediated by similar processes. While Acartiid copepods have relatively small energetic reserves compared to other calanoids (e.g., the large lipid reserves of *Calanus* species; Lee et al., 2006), these energetic reserves may still buffer against deleterious effects of starvation over several days (Hirahara & Toda, 2018). The 3‐day buffer against decreases in thermal limits that we observed in this study contrasts the rapid drop‐off of egg production and respiration rates in starved *A. tonsa* females (Kiørboe et al., 1985; Parrish & Wilson, 1978; Thor, 2003). This may indicate different biochemical bases, or that environmental tolerance and survival is prioritized in energetic allocation. It is important to address the physiological basis for the observed patterns and disentangle cause and consequence (MacMillan, 2019). One approach may be to examine how patterns in respiration rates (Kiørboe et al., 1985) and heat shock protein expression (Nguyen et al., 2017) change during starvation and heat stress (both individually and when combined; Dinh et al., 2016).

Developing an understanding of how changing environmental factors affect the thermal performance of copepods is crucial due to their role as the linkage between primary producers and larger consumers in aquatic foodwebs. Moreover, the factors affecting the physiological limits of *A. tonsa* specifically are important to understand given the key role this widely distributed species plays in coastal communities (Turner, 1981, 2004). It is well known that phenotypic plasticity and genetic differentiation both affect the upper thermal limits in this species (Sasaki & Dam, 2019), potentially facilitating its wide distribution. Factors that limit physiological performance in this species are also important to understanding for predicting invasion risk across temperate and subtropical estuaries, where anthropogenic introduction of this species may alter community dynamics (Aravena et al., 2009; Svetlichny & Hubareva, 2014). In low latitudes, high temperatures may limit copepod productivity (Doan et al., 2019). Additional experiments are needed to understand how starvation effects on upper thermal limits may change across temperature gradients in order to understand whether starvation may amplify these effects on productivity, thus establishing a boundary to expansion. However, assuming an energetic basis to the reduction in thermal limits observed, we might expect to see larger, more rapid decreases in thermal limits when high temperatures and starvation co‐occur, potentially inhibiting invasion of warm, oligotrophic regions. Our results also suggest that work examining how starvation affects the capacity of plasticity (either short‐term acclimation or developmental plasticity) to increase thermal limits is also needed.

Another open question is to what extent sex‐specific differences in starvation sensitivity or developmental effects of food limitation may influence population dynamics. Females in this species are known to be both more resistant to starvation (Finiguerra et al., 2013) and to have higher thermal limits than males (Sasaki et al., 2019). Increased male sensitivity to starvation relative to females (resulting in a more rapid or a larger decrease in thermal limits) may amplify negative population demographic effects of co‐occurring food limitation and exposure to high temperatures. The effects of developing at different food levels may also influence these dynamics. Developmental patterns may be strongly influenced by food limitation, with increases in total development time, and decreases in adult size (Hirst et al., 2003; Vidal, 1980). This may dampen starvation effects on thermal limits simply because smaller adults typically require less food to maintain basal energetic demands. This may result in an increased capacity to maintain thermal limits under starvation in individuals that completed development at low food levels. While ontogenetic patterns in respiration rates are resilient to heat stress in *A. tonsa* (Holmes‐Hackerd et al., 2023), the effects of food limitation during development may have carry‐over effects on adult respiration and energetic requirements that may modify these predictions. These carry‐over effects are, however, still unknown in copepods, but work in insect systems suggests that food limitation during the larval stage affects the energy status of the adult (Dinh et al., 2016).

Regardless of the mechanistic basis, our results highlight the importance of understanding temporal scales of physiological variation for predictions about vulnerability. Over short timescales, *A. tonsa* females are able to maintain thermal limits under starvation. This suggests that starvation is unlikely to increase the vulnerability of natural populations to acute events like heatwaves. Longer timescale decreases in food availability, however, may have dire consequences for populations in a warming ocean. Although research on large‐scale changes in phytoplankton biomass due to climate change lacks consensus (Archibald et al., 2022; Boyce et al., 2010; Taucher & Oschlies, 2011), broad decreases in vertical mixing associated with the intensified thermal stratification and shifts in the phenology of the spring bloom to earlier in the year (Sommer & Lengfellner, 2008) may drive an increase in small, motile phytoplankter and heterotrophic microzooplankton in many regions (Winder & Sommer, 2012). Primarily herbivorous copepods may, therefore, be particularly vulnerable to warming compared to omnivorous species like *A. tonsa*, which may avoid starvation and the accompanying reduction in thermal limits by feeding on heterotrophic microzooplankton. Given the large contributions of herbivorous species to carbon flux in marine systems (Pinti et al., 2023), these shifts in community composition driven by differential environmental sensitivity would have important biogeochemical consequences.

## AUTHOR CONTRIBUTIONS

**Matthew C. Sasaki:** Conceptualization (lead); data curation (lead); formal analysis (lead); investigation (equal); methodology (lead); project administration (lead); supervision (lead); visualization (lead); writing – original draft (supporting); writing – review and editing (equal). **Gaia Rueda Moreno:** Data curation (supporting); formal analysis (supporting); investigation (equal); methodology (supporting); writing – original draft (lead); writing – review and editing (equal).

## CONFLICT OF INTEREST STATEMENT

None declared.

### OPEN RESEARCH BADGES

This article has earned Open Data and Open Materials badges. Data and materials are available at https://doi.org/10.5281/zenodo.8057948.

## Data Availability

All data and scripts used in the analyses presented here are available in a Zenodo repository (https://doi.org/10.5281/zenodo.8057948). Scripts are also actively maintained on the associated GitHub repository.
